# Quality Measurement in Shanghai From a Global Perspective; A Response to Recent Commentaries

**DOI:** 10.34172/ijhpm.2024.8491

**Published:** 2024-04-28

**Authors:** Alon Rasooly, Yancen Pan, Zhenqing Tang, Jiangjiang He, Ruitai Shao, Moriah E. Ellen, Orly Manor, Shanlian Hu, Nadav Davidovitch

**Affiliations:** ^1^School of Public Health, Ben-Gurion University of the Negev, Beer Sheva, Israel.; ^2^Department of Epidemiology, Fielding School of Public Health, University of California – Los Angeles, CA, USA.; ^3^Shanghai Health Development Research Center, Shanghai, China.; ^4^Department of Chronic Disease & Multimorbidity, School of Population Medicine and Public Health, Chinese Academy of Medicine Science/Peking Union Medical College, Beijing, China.; ^5^Braun School of Public Health and Community Medicine, Hebrew University, Jerusalem, Israel.; ^6^School of Public Health, Fudan University, Shanghai, China.

## Background

**Research Highlights**

► China’s top-down directive complements the “why” of quality measurement, which seeks to achieve domestic standardization.
► Many countries and international organizations have adopted diabetes quality indicators using scientific evidence that ties measurements to health outcomes.
► Implications for China include incorporating relevant social determinants of health when measuring and communicating the quality of care.

The centrality of high-quality primary care is universally acknowledged as pivotal to the healthcare system’s performance and the well-being of individuals in society.^1^ As countries strive to strengthen their primary care foundations, developing and applying tools for measuring performance have become crucial, with some implementing strategies like “pay-for-performance.”^2^ China has profoundly transformed its health system over the past 25 years since its healthcare reform, particularly its focus on strengthening primary care.^3^ This reform has witnessed the implementation of performance indicators for diabetes linked to primary care providers’ remuneration.^4^ However, the outcomes yielded mixed results with a minor effect on mitigating health inequities.^5^ In light of these dynamics, our qualitative study^4^ investigated performance measurements in primary diabetes care in Shanghai, contributing to ongoing discussions about the direction of primary care quality in the Chinese context.

 This response commentary replies to four insightful perspectives that further enrich the discourse initiated by our work.^6-9^ Each commentary offered a unique lens, exploring facets such as the implications of China’s healthcare reforms, the persistent challenges in primary care services, the global context of quality measurement, and the intricate considerations involved in defining and assessing high-quality care. Collectively, the four commentaries^6-9^ provided a comprehensive and multifaceted backdrop for understanding the complexities of primary diabetes care, ensuring a robust foundation for ongoing dialogues and potential avenues for improvement. In addressing these commentaries, we aim to contribute to the ongoing conversation surrounding primary care measurement, incorporating diverse viewpoints to foster a more nuanced and holistic understanding. Inspired by Matulis and McCoy’s commentary,^6^ we reviewed the quality measurement in Shanghai and compared it to the international experience using a set of four key questions (Table).

**Table:** Key Questions on Quality Measurement in Shanghai Compared With Other Countries

**Key Question**	**Shanghai (China)**	**Alternatives**	**International Examples**
Why is quality measured?	Standardizing local practices and processes in line with the policy formulated nationally.	Standardizing between national and international indicators, reducing health inequities, and aligning care with patients’ expectations and needs.	Saudi Arabia’s Diabetes Registry,^10^ Israel’s QICH program,^11^ and PROMs in Sweden’s NDR^12^
Who defines quality?	Policy-makers and scholars affiliated with China’s National Health Commission.	Multiple stakeholders’ consortiums and patients’ perspectives.	ICHOM,^13^ Germany’s RKI,^14^ Sweden’s NDR^12^
What criteria are used to select quality indicators?	Alignment with national guidelines, feasibility, and test affordability.	Alignment with international standards, evidence-informed, association with health outcomes.	Diabetes programs in the OECD,^15^ European Region,^16^ UAE,^17^ Malaysia^18^
How do quality indicators lead to improvement?	External motivators, pay-for-performance, physician accountability.	Internal motivators, transparency, network accountability.	British QOF,^19^ Israel QICH,^20^ Minnesota (USA)^21^

Abbreviations: ICHOM, International Consortium on Health Outcome Measurement; NDR, National Diabetes Register; OECD, Organisation for Economic Co-operation and Development; PROMs, patient reported outcome measures; QICH, Quality Indicators in Community Healthcare; QOF, Quality and Outcomes Framework; UAE, United Arab Emirates; RKI, Robert Koch Institute.

## The Why, What, Who, and How of Quality Measurement

**Figure 1:**
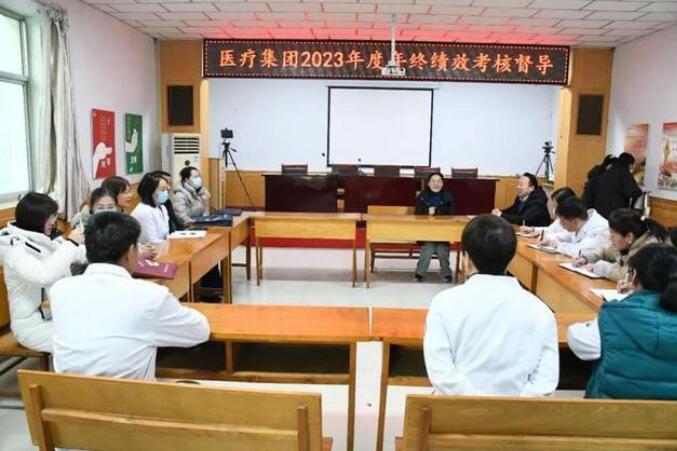
A Rural Township Health Center Performance Appraisal Meeting in Ruicheng County, Shanxi Province, China. The board reads: “The Medical group’s performance appraisal evaluation for 2023”. Source link.

 The first, “*Why*” question, sets out to uncover the motivations and interests for developing and maintaining the quality measurement program. According to our article,^4^ quality measurement programs in China are centrally planned and provide a means for standardizing local practices and processes in line with the policy formulated nationally in Beijing. This finding agrees with Xu’s commentary,^9^ who noted that “a typical scene in a rural township health center is a big board glued to the wall of the director’s office. On the board is a large table including a range of performance indicators… well aligned if not exactly the same as the national performance evaluation guideline.” Indeed, a rapid online search elicited images of meetings from primary health centers across China, showing managers and health professionals reviewing their centers’ performance reports (Figure 1 and Supplementary file 1). These vivid descriptions resonate with our article’s findings and strengthen our research’s generalizability to other parts of China.

 Overseas, quality measurement programs in other countries were developed for different reasons than in China. In Saudi Arabia, an Interactive Diabetes Registry was established to address the diabetes epidemic by improving data for prevention, disease management, and research.^10^ In Israel, the Quality Indicators in Community Healthcare (QICH) program was developed to evaluate the treatment level relative to national and international indicators.^11^ In addition, some countries such as Sweden have emphasized the perspective of individuals living with diabetes and have developed a set of patient-reported outcome measures (PROMs) to be incorporated into the country’s National Diabetes Register.^12^ Implementing PROMs in China’s context aligns with Jian et al commentary,^8^ which called for “using indicators that are more relevant to residents’ health.”

 Regarding the question of “*Who* defines quality,” our study^4^ suggests that national-level policymakers define the quality of care for Shanghai and other regions in a top-down manner. To be more specific, health policies such as the “National Basic Public Health Service Standards”^22^ are formulated by the National Health Commission and its affiliated scholars. China’s top-down directive complements the “why” of quality measurement, which seeks to achieve domestic standardization.^4^ It is worth noting that countries differ on the issue of who defines quality measurement. For example, Germany’s Robert Koch Institute (RKI) established a national diabetes surveillance system^23^ in line with the approach adopted by the World Health Organization (WHO).^24^ To ensure international compatibility, a panel of experts from the United States, Denmark, Canada, Italy, and Scotland assessed the potential indicators to be implemented in Germany and presented their insights at an international scientific workshop convened at RKI.^14^ Beyond high-income countries, a recent review in low- and middle-income countries shows that actions to enhance citizen engagement, like improving direct communication between users and providers, effectively increase active citizen involvement in service delivery.^25^ This leads to better access and quality, especially for services with direct citizen-provider interaction, such as healthcare.^25^

 When reviewing “*What* criteria are used to select quality indicators,” it appears that in China, alignment with national guidelines, feasibility, and test affordability plays a central role. The third edition of the National Basic Public Health Service Standards^22^ exemplifies this by listing two indicators for diabetes: (1) *Standardized management rate –* health management of patients in accordance with regulatory requirements, and (2) *Glycemic control rate – *the ratio of people who reached the fasting blood glucose standard (<7 mmol/L) at the latest follow-up divided by the diabetes population managed for a given year. Therefore, the first indicator effectively binds primary healthcare providers to the National Health Commission’s regulatory requirement. Simultaneously, the second indicator roots the foundations of diabetes quality measurement in China on the ever-fluctuating and inexpensive fasting blood glucose test rather than the evidence-informed albeit more expensive glycated hemoglobin (HbA1C) test.^26^ In comparison, many countries and international organizations have adopted diabetes quality indicators using scientific evidence that ties measurements to health outcomes.^11,16-18,27^ This includes poor glycemic control using HbA1C threshold values (>9%), which predicts the risk of stroke and myocardial infarction,^28^ and microalbuminuria, which forecasts impending renal failure.^29^

 Regarding the mechanism connecting measurement and improvement, or the *how* question, our article suggests that external motivators, particularly pay-for-performance incentives, and physician accountability, play a major role in China’s context.^4^ While such incentives are prevalent, they have been criticized for encouraging physicians not to enroll complicated patients in Taiwan,^30^ rewarding the wrong providers in Canada,^31^ and were associated with a decline in performance after incentive removal in the United Kingdom.^19^

 In Minnesota (USA), researchers examined the impact of public reporting and the progression of diabetes care quality, discovering that clinics participating in early reporting consistently maintained higher quality standards than those reporting later throughout the studied years.^21^ In Israel’s QICH program, measurements are transparently reported every two years for the four competing health management organizations.^20^ Each of the health management organizations is held accountable on the network level, while physicians have an internal motivation to provide excellent care for their patients. Physicians can compare their performance to the network average, but there are no “carrots and sticks” for scoring above or below.^20^ Evidence showed that patients with diabetes adhering to the QICH quality measurements experienced a lower risk of cardiac morbidity, validating the program’s effectiveness.^32^

## Addressing Health Inequities

 In the commentaries by Neumann^7^ as well as by Matulis and McCoy,^6^ a question was raised about the relationship between quality measurements, health inequities, and the social determinants of health in the studied context. In our research, we found that measurements introduced nationally, such as the *glycemic control rate*, were presented without stratified reporting based on social determinants of health, furthering away the potential to abate health inequities.^4^

 Evidence from China and abroad highlights the strong association between social determinants of health and diabetes-associated health outcomes.^5,33–35^ According to a nationally representative study by Sun et al^5^ the proportions of patients with diabetes-related and recurrent hospitalizations in western China were higher than those in the more affluent eastern region. Furthermore, as noted by Xu’s commentary,^9^ data from China’s National Center for Cardiovascular Diseases shows widening gaps between urban and rural cardiovascular disease mortality rates,^36^ an outcome tightly linked with glycemic control.^28^

 Numerous examples of countries have evaluated the quality of diabetes care with stratification to social determinants.^32,37-39^ In the United States, researchers assessed racial and ethnic differences in attaining diabetes measurements following the expansion of Medicaid eligibility in 2014. Using an HbA1C cutoff of 9%, they have observed improvement in glycemic control, particularly among the Black and Hispanic populations, which tended to increase with implementation time.^40^ The Israeli QICH program highlighted health inequities, including in diabetes care, by using exemptions from National Insurance Institute payments as an indicator of low socioeconomic status and in later years by socio-economic position (SEP) based on geographical areas.^20^ Stratification of data using SEP uncovered a near-to-three times difference in the rate of poor glycemic control (HbA1C >9%) among people from the lowest SEP quartile (16.2%) compared with people from the highest quartile (5.5%).^20^ Presenting evidence of health inequities to decision-makers can empower national policy changes to mitigate socioeconomic gaps in access to diabetes care.^41^

**Figure 2:**
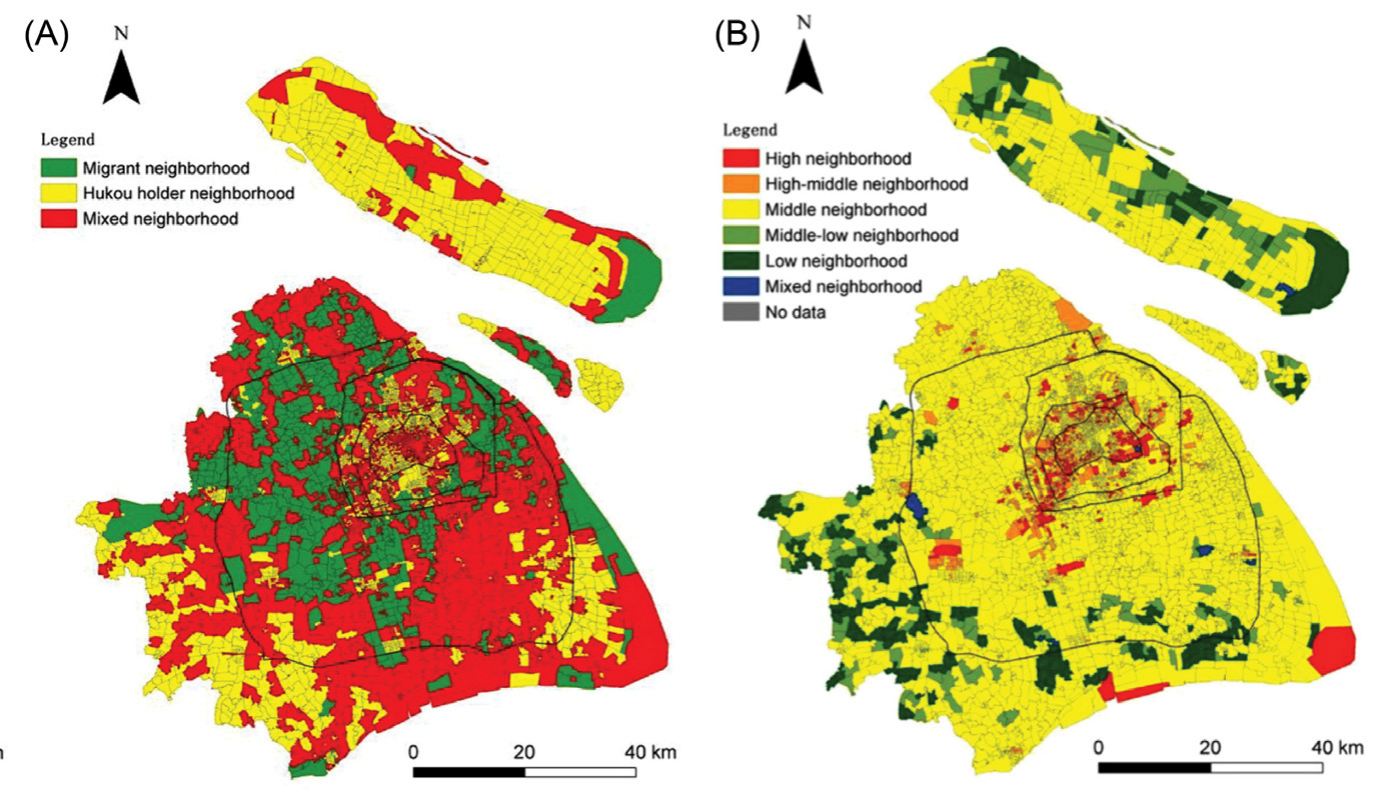
Socio-spatial Analysis of Neighborhood Types by Residency Composition (A) And Educational Composition (B) in Shanghai, 2010. Figure from an article by Shen and Xiao,^44^ adapted with permission from the corresponding author. Note: The term “Hukou” denotes registration of an individual as a permanent resident of an area.

 Implications for China include incorporating relevant social determinants of health when measuring and communicating the quality of care. On the macro level, the urban-rural and eastern-western divides illuminate some of the social inequities in China and have been reported in several studies on diabetes.^5,42^ However, we suggest that researchers and policymakers should take a step forward toward assessing health inequities in a higher resolution. For example, stratifying the quality of care according to residency status can uncover health inequities among internal migrants, who tend to utilize fewer health services compared with permanent residents in China’s megacities.^43^ In Shanghai, Shen and Xiao^44^ conducted a socio-spatial analysis using residential-level data to uncover high-status and migrant neighborhoods and further stratify communities according to their educational composition. Incorporating socioeconomic status variables from the neighborhood level into the evaluation of diabetes quality in Shanghai and other megacities across China can be a powerful tool for mitigating health inequities (Figure 2).

## Conclusion

 Our analysis of diabetes care quality in Shanghai highlights a centralized approach to defining and implementing measurements, distinct from international practices. While China’s emphasis on central planning aims at domestic standardization, our comparison with global experiences emphasizes the varied motivations and criteria for quality measurement. The study also draws attention to health inequities, urging China to consider relevant social determinants, such as residency status and socioeconomic variables, for a more nuanced assessment and mitigation of inequities in diabetes care.

## Acknowledgments

 We express our sincere gratitude to the researchers who provided valuable insights and comments on our manuscript; their contributions sparked a thought-provoking discussion, elaborated upon in this response commentary.

## Ethical issues

 Not applicable.

## Competing interests

 Authors declare that they have no competing interests.

## Supplementary files

Supplementary file 1. A Community Health Center Performance Appraisal Meeting in Shijingshan District, Beijing, China.
