# Supplementary material for: Quality Measurement in Shanghai From a Global Perspective; A Response to Recent Commentaries
Source: Int J Health Policy Manag. 2024 Apr 28;13:8491. doi: 10.34172/ijhpm.2024.8491 (PMC11270598; doi:10.34172/ijhpm.2024.8491)

Article title: Quality Measurement in Shanghai From a Global Perspective; Response to Recent Commentaries

Journal name: International Journal of Health Policy and Management (IJHPM)

Authors' information: Alon Rasooly[1]*, Yancen Pan[2], Zhenqing Tang[3], Jiangjiang He[3], Ruitai Shao[4], Moriah E. Ellen[1], Orly Manor[5], Shanlian Hu[6], Nadav Davidovitch[1]

[1]School of Public Health, Ben-Gurion University of the Negev, Beer Sheva, Israel.

[2]Department of Epidemiology, Fielding School of Public Health, University of California – Los Angeles, Los Angeles, CA, USA.

[3]Shanghai Health Development Research Center, Shanghai, China.

[4]Department of Chronic Disease & Multimorbidity, School of Population Medicine and Public Health, Chinese Academy of Medical Sciences & Peking Union Medical College, Beijing, China.

[5]Braun School of Public Health and Community Medicine, Hebrew University, Jerusalem, Israel.

[6]School of Public Health, Fudan University, Shanghai, China.

***Correspondence to:** Alon Rasooly; Email: rasooly@post.bgu.ac.il

Citation: Rasooly A, Pan Y, Tang Z, et al. Quality measurement in shanghai from a global perspective; response to recent commentaries. Int J Health Policy Manag. 2024;13:8491. doi:10.34172/ijhpm.2024.8491

Supplementary file 1. A Community Health Center Performance Appraisal Meeting in Shijingshan District, Beijing, China

The board reads: "Community health performance assessment implementation plan seminar for 2019". Source link.

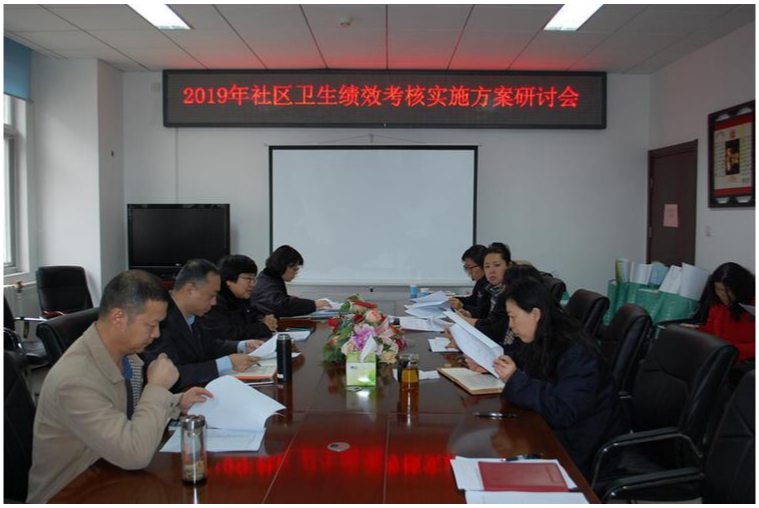

Supplement: Supplementary file 1 — A Community Health Center Performance Appraisal Meeting in Shijingshan District, Beijing, China. [file ijhpm-13-8491-s001.pdf]
